# Delivery fidelity of the REACT (REtirement in ACTion) physical activity and behaviour maintenance intervention for community dwelling older people with mobility limitations

**DOI:** 10.1186/s12889-022-13496-z

**Published:** 2022-06-03

**Authors:** Rosina Cross, Colin J. Greaves, Janet Withall, W. Jack. Rejeski, Afroditi Stathi

**Affiliations:** 1 Department for Health, University of Bath, Claverton Down, Bath, BA2 7AY UK; 2 School of Sport, Exercise and Rehabilitation, Sciences, University of Birmingham, Egbaston, Birmingham, B15 2TT UK; 3 Department of Health and Exercise Science, Wake Forest University, Worrell Professional Centre, 2164B, PO Box 7868, Winston-Salem, NC 27109 USA

**Keywords:** Process evaluation, Behaviour change, Physical activity, Behavioural intervention, Older adults, Randomised controlled trial, Behaviour change techniques

## Abstract

**Background:**

Fidelity assessment of behaviour change interventions is vital to understanding trial outcomes. This study assesses the delivery fidelity of behaviour change techniques used in the Retirement in ACTion (REACT) randomised controlled trial. REACT is a community-based physical activity (PA) and behaviour maintenance intervention to prevent decline of physical functioning in older adults (≥ 65 years) at high risk of mobility-related disability in the UK.

**Methods:**

The delivery fidelity of intervention behaviour change techniques and delivery processes were assessed using multi-observer coding of purposively sampled in-vivo audio recordings (n = 25) of health behaviour maintenance sessions over 12-months. Delivery fidelity was scored using a modified Dreyfus scale (scores 0–5) to assess competence and completeness of delivery for each technique and delivery process. “Competent delivery” was defined as a score of 3 points or more for each item. Examples of competent intervention delivery were identified to inform recommendations for future programme delivery and training.

**Results:**

The mean intervention fidelity score was 2.5 (SD 0.45) with delivery fidelity varying between techniques/processes and intervention groups. Person-centred delivery, Facilitating Enjoyment and Promoting Autonomy were delivered competently (scoring 3.0 or more). There was scope for improvement (score 2.0—2.9) in Monitoring Progress (Acknowledging and Reviewing), Self-Monitoring, Monitoring Progress (Eliciting Benefits of Physical Activity), Goal Setting and Action Planning, Modelling, Supporting Self-Efficacy for Physical Activity and Supporting Relatedness. Managing Setbacks and Problem Solving was delivered with low fidelity. Numerous examples of both good and sub-optimal practice were identified.

**Conclusions:**

This study highlights successes and improvements needed to enhance delivery fidelity in future implementation of the behavioural maintenance programme of the REACT intervention. Future training of REACT session leaders and assessment of delivery fidelity needs to focus on the delivery of Goal setting and Action Planning, Modelling, Supporting Relatedness, Supporting Self-Efficacy for Physical Activity, and Managing Setbacks/ Problem Solving.

**Supplementary Information:**

The online version contains supplementary material available at 10.1186/s12889-022-13496-z.

## Background

Physical activity (PA) interventions that incorporate behaviour change strategies or techniques (BCTs) have been shown to be effective at increasing physical activity levels but reported effectiveness of these interventions varies significantly [1–5]. Behaviour change interventions are complex, typically employing numerous components that are intrinsically linked and are difficult to design, implement, evaluate, and replicate [6, 7]. Assessments of intervention fidelity provide insight into the proposed mechanisms of behaviour change, better understanding of how change takes place, why changes may not be observed, and whether any positive change BCTs can be replicated [7–9]. Intervention fidelity is the extent to which an intervention is delivered as intended and monitoring it can enhance an intervention’s internal and external validity [7–9].

Without an understanding of fidelity, an intervention could produce significant results, but it would be impossible to say whether this was a function of the intervention content or the addition of unknown content [7, 10]. Alternatively, an intervention may produce non-significant results, but without understanding fidelity, it is difficult to know if this is due to an ineffective intervention or a failure to deliver its active components [7, 10]. As a result, an intervention that may have been effective if it had been delivered correctly may be misleadingly deemed ineffective [7, 10].

A treatment fidelity framework developed by the behaviour change consortium (BCC) outlines five domains of treatment fidelity [8, 9]. These are; study design, provider training, treatment delivery, treatment receipt, and treatment enactment [8, 9]. Intervention delivery fidelity assesses whether the intervention was delivered as designed; i.e., did the person/s delivering the intervention adhere to or deviate from intervention protocol, and if so, how [8]? This concept overlaps with the MRC process evaluation component of implementation [7]. A recent systematic review suggested that there is a lack of robust fidelity assessment within the field of physical activity research since objective measures of fidelity are rare [11].

The current study assesses the delivery fidelity of the Retirement in ACTion (REACT) study, a pragmatic, randomised controlled trial of a community-based physical activity and behaviour maintenance intervention to prevent decline of physical functioning in older adults [12]. In addition, it identifies examples of good practice to inform future training in the delivery of the REACT intervention and other community-based, active ageing programmes.

## Methods

### Study design

The REACT study was a pragmatic multi-centre, two arm, single blind, parallel-group, randomised controlled trial (RCT) with an internal pilot phase, incorporating comprehensive process and economic evaluations [13]. Intervention sessions were delivered over 12 months, in two phases (adoption (week 1 – 24) and maintenance (Week 24 – 52)). Exercise sessions were twice weekly for the first 12 weeks, then weekly up to 52 weeks [13]. A series of health behaviour maintenance sessions were delivered weekly from weeks 9 to 24, then monthly from weeks 24 to 52. These sessions included BCTs and processes to a) enhance motivation; b) help participants set realistic goals for sustainable PA; c) identify possible barriers and ways to overcome them; d) encourage social support; and e) support participants to use BCTs (e.g. self-regulation techniques like self-monitoring) to maintain physical activity behaviour change [13].

All REACT sessions were led by a REACT session leader, exercise professionals trained to deliver exercise for older adults in a safe manner. REACT session leaders were all qualified to at least Level 3 (Exercise Referral Diploma or equivalent) and received specific training in the delivery of the REACT health behaviour maintenance sessions. This training focused on intervention delivery methods, communication styles, the REACT logic model, and BCTs [13]. Training also included detailed session plans and a REACT session manual developed by the intervention designers to ensure consistency and fidelity of intervention delivery [13]. A sample REACT health behaviour maintenance session plan can be found in Additional file 1. There was no formal assessment of session leader competence to deliver REACT health behaviour maintenance session content at the end training period [13].

**Fig. 1:**
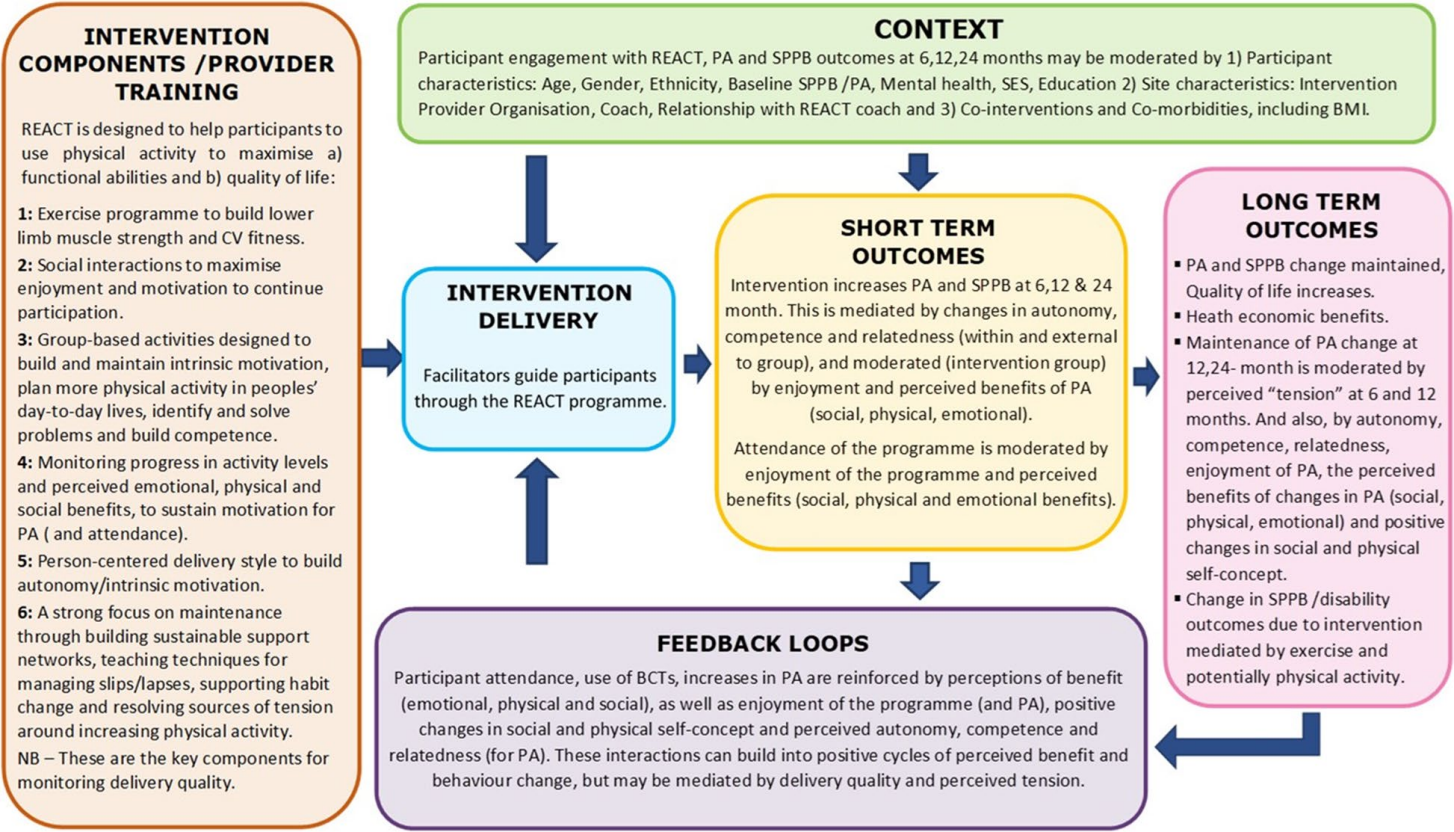
REACT Intervention Logic Model

A process evaluation of the REACT intervention was designed to test the REACT Logic Model which illustrates intervention processes and proposes mechanisms of impact (Fig. 1). This process evaluation included an assessment of the delivery fidelity of the intervention, which was designed to inform further refinement of the intervention and future implementation, as well as to generate data to help interpret the trial findings on intervention effectiveness and their likely generalisability [13]. The intervention fidelity evaluation was an observational study based on observer rating of in-vivo audio recordings of the REACT health behaviour maintenance sessions. Examples of good practice in intervention delivery were identified to inform recommendations for future programme delivery and training.

### Theoretical framework underpinning the REACT intervention

The theoretical basis of REACT health behaviour maintenance sessions draws on two overlapping psychological theories; Social Cognitive Theory (SCT) [14] and Self-Determination Theory (SDT) [15, 16] to provide the BCTs and processes for supporting behaviour change. These included key behaviour change processes from SCT (e.g. Self-Efficacy and Modelling) alongside BCTs such as, Monitoring Progress (acknowledging/ reviewing and eliciting the benefits of PA), Self-Monitoring, Managing Setbacks and Problem Solving, Goal Setting and Action Planning [14] classified within the Behaviour Change Taxonomy [17] and key motivational processes from SDT (autonomy, competence and relatedness) [15, 16].

#### Sampling

Recordings of the REACT health behaviour maintenance sessions were purposively sampled to include a diverse sample of sessions based on, a) coverage of key BCTs and processes included in the session plans (the BCTs or processes present in each sampled session are shown in Table 1), b) intervention provider (organisations responsible for delivering the intervention sessions) and intervention session leader, and c) the inclusion of sessions relating to key transition points in the intervention. Key transition points included;

- The first health behaviour maintenance session (Week 9)
- The transition from two exercise sessions a week to one exercise session a week (Week 13) where participants were encouraged to source physical activity opportunities independently from the REACT programme
- The transition from one health behaviour maintenance session per week, to one per month (Week 24)
- A typical monthly session between week 24 and 52: Week 28 was sampled, which focused on re-visiting and reinforcing motivation and goal setting for home and neighbourhood-based physical activities and exercise
- The final two health behaviour maintenance sessions (Week 48 and Week 52). These sessions focused on preparing people to be active beyond the end of the REACT programme

**Table 1:** Behaviour Change Techniques used in each sampled health behaviour maintenance session

Intervention Behaviour Change Technique	Intervention Weeks Sampled									Number of intended sessions
	**9**	**12**^**a**^	**13**^**b**^	**16**	**20**	**24**^**c**^	**28**	**48**^**d**^	**52**^**e**^	
**Person-Centred Delivery**	X	X	X	X	X	X	X	X	X	9
**Facilitating Enjoyment**	X	X	X	X	X	X	X	X	X	9
**Monitoring progress (Acknowledging and Reviewing)**	X	X	X	X	X	X	X	X	X	9
**Monitoring Progress (Eliciting benefits of PA)**	X	X	X	X	X	X	X	X	X	9
**Self-Monitoring**	X	X	X	X	X					5
**Managing Setbacks and Problem Solving**	X	X	X	X	X	X	X	X	X	9
**Goal setting and action planning**	X	X	X	X	X	X	X	X	X	9
**Modelling**	X	X								2
**Promoting Autonomy**	X	X	X	X	X	X	X	X	X	9
**Supporting Self-efficacy for PA**	X	X	X	X	X	X	X	X	X	9
**Supporting Relatedness**	X	X	X	X	X	X	X	X	X	9

^a^Health behaviour maintenance sessions start

^b^Exercise sessions drop from twice a week to once a week

^c^End of the adoption phase/start of maintenance phase

^d^Health behaviour maintenance sessions drop to once a month

^e^Last REACT session

### Measures

#### Fidelity checklist

Delivery fidelity (content and quality) was monitored via the application of a delivery fidelity checklist applied to audio-recordings of health behaviour maintenance sessions. The 11-item checklist was designed by the first author (RC) and REACT research team members who developed the health behaviour maintenance sessions (CG, AS) to a) assess key intervention processes and BCTs illustrated in the REACT Logic Model (Fig. 1), and b) measure the extent to which REACT session leaders delivered the intervention BCTs and processes as intended. Each checklist item reflected a key BCT or process and was defined in terms of a set of intended techniques or practices (Table 2). Audio recording, using an Olympus VN-741PC digital voice recorder, was deemed to be more feasible and less intrusive than using video recordings [18].

**Table 2:** REACT Intervention BCTs and processes included in the intervention fidelity analysis

Intervention Behaviour Change Techniques and processes	Intended delivery of techniques
Person centred delivery Communication should be participant focused, maximising participant autonomy (Intervention Process)	Use of open-ended questions
	Affirmations for positive behaviours, recognising efforts to change, as well as their autonomy to make changes
	Reflective listening (actively engage with participant, empathise, reflect emotional state, summarise discussion)
	Summaries can be used to reinforce participant choices and acknowledging participant effort or success
	Using the Ask-Tell-Discuss technique to exchange /deliver key information
Facilitating Enjoyment (Intervention process)	Using the techniques associated with Person-centred delivery (as above), session leaders should encourage and reinforce enjoyment of social interactions within the group by making the social interactions positive, supportive and enjoyable, rather than embarrassing and awkward
Monitoring Progress (Acknowledging and Reviewing) (BCT (Self-Regulation))	Using the techniques associated with Person-centred delivery (as above), session leaders should regularly acknowledge and review the progress of group members in terms of their physical activity levels
Monitoring Progress (Eliciting and reinforcing the benefits of Physical Activity) (BCT (Self-Regulation))	Using the techniques associated with Person-centred delivery (as above) facilitator should encourage discussion on the emotional, social and physical benefits of physical activity
Self – Monitoring (BCT (Self-Regulation))	Using techniques associated with Person-centred delivery (as above) session leaders should encourage participant self-monitoring, acknowledge participant attempts to self-monitor as well as any progress made with self-monitoring
Managing Setbacks and Problem Solving (BCT (Self-Regulation))	Using techniques associated with Person-centred delivery (as above) session leaders should encourage discussion on setbacks participants have experienced and encourage problem solving. This should involve reviewing progress with planned changes and targets set out in action plans as well as celebrating and reinforcing any successes, while reframing and normalising setbacks. Problems should be broken down, and the sustainability of coping plans and the support others can provide should also be considered
Goal setting and Action Planning (BCT (Self-Regulation))	Using techniques associated with Person-centred delivery (as above) session leaders should work with the participants to agree on action plans, including; negotiating of goals, goal setting and identifying any barriers that may arise. Session leaders should acknowledge participants perspective and encourage participant input throughout
Modelling (Intervention Process (Social Cognitive Theory))	Using techniques associated with Person-centred delivery (as above) session leaders should give participants the opportunity to observe others engaging appropriately with the programme
Promoting Autonomy (Intervention Process (Self-Determination Theory))	Using techniques associated with Person-centred delivery (as above) session leaders should encourage pro-active involvement in the classes and discussion. Create opportunities for participant input, while acknowledging participant perspectives, encouraging participants to be the driver of change and develop a sense of control
Supporting Self-Efficacy for PA (Intervention Process (Self-Determination Theory & Social Cognitive Theory))	Using techniques associated with Person-centred delivery (as above) session leaders should encourage participants, identify and break down barriers to change, set achievable goals /encourage gradual progress, give appropriate and constructive feedback and check for understanding. Encourage problem-solving and ascertain participant confidence and skills so these can be built upon throughout the intervention sessions
Supporting Relatedness (Intervention Process (Self-Determination Theory)	Using techniques associated with Person-centred delivery (as above) session leaders should fulfil participants needs for relatedness (social engagement/ acceptance, approval of one’s behaviour and giving support to others). This can be promoted by encouraging engagement in physical activity, where there are opportunities for positive social interactions as well as highlighting physical activity as a social opportunity

The rating scale applied to the REACT intervention fidelity checklist is based on a six-point Dreyfus scale [19] widely used for assessing competence in the delivery of clinical consultations. This is used to measure the session leaders’ adherence to the use of intervention BCTs and processes, as well as the skill with which they are delivered (Table 3). The Dreyfus Scale extends from (0) indicating that the facilitator did not deliver the intervention BCT appropriately – either it was badly executed or not executed enough—to (5) indicating the BCT is delivered exceptionally well (Table 3) [19]. Key features of each BCT and detailed scoring instructions for using the fidelity measure to assess delivery fidelity of the REACT health behaviour maintenance sessions can be found in Additional file 2.

**Table 3:** The adapted Dreyfus scale for scoring REACT delivery fidelity

Competence Level	Scoring	Examples	Delivery Fidelity Categories
Absence	0	Absence of feature and/ or highly inappropriate performance	Low fidelity
Novice	1	Minimal use of feature and /or inappropriate performance	Low fidelity
Advanced Beginner	2	‘Scope for improvement’, alongside numerous minor and some major inconsistencies and/or problems	Scope for Improvement
Competent	3	Competent, good features but some minor inconsistencies or problems	Competent
Proficient	4	Very good features, but minimal inconsistencies or problems	Proficient
Expert	5	Excellent features, no problems or inconsistencies	Expert

### Scoring and reliability

To reduce subjectivity in the scoring process, two coders (RC and CG) independently coded sessions (*n* = 10), followed by discussions to resolve any discrepancies. If discrepancies in scoring between coders exceeded more than 1 point on the 6-point Likert scale, the sessions were discussed and re coded. The remaining sessions were then coded by one coder (RC). The sample frame for sessions to be double-coded was based on diversity and achieving a representative subsample, based on variation in session leaders, sites, locations and weeks sampled. We adopted this “iterative calibration” approach, whereby the coders compared notes and ideas after coding every 2–3 sessions. This led to convergence of the coding approach with little or no disagreement after 4 iterations. As advocated by other assessments of delivery fidelity [11, 20, 21] the coding of delivery fidelity was anchored to the key heuristic that a score of 3 was considered to represent “competent delivery” – i.e. delivery that was considered sufficient to deliver the intended BCTs or processes of the intervention. The range of scores and their interpretation is provided in Table 3.

### Examples of REACT delivery practice

While coding for intervention delivery fidelity, researchers noted down examples of theorised and non-theorised intervention processes being delivered in practice. These examples were time stamped and tabulated to enable identification of examples of good practice and delivery needing improvement (Additional file 4).

### Analysis

For each of the sampled REACT groups, scores representing the delivery fidelity for each fidelity checklist item were recorded on a spreadsheet.

Fidelity checklist scores were summarised by calculating either a mean or a maximum score for each item across all coded sessions. Mean scores were calculated for items representing delivery processes or BCTs that were intended to be delivered in every session (e.g. Person-Centred Delivery and Managing Setbacks and Problem-Solving). Maximum scores were used for items representing delivery processes of BCTs that were intended to be delivered in only some of the sessions (e.g. Self-monitoring and Modelling). A table summarising which checklist items were attributed mean or maximum scores can be found in Additional file 3. A mean item score (combining all 11 items) was then calculated for each group, as well as an overall delivery fidelity score for each intervention group and the intervention as a whole (the mean of all checklist item scores).

## Results

From an intended sample of 54 purposively sampled audio-recordings, 25 (46%) were suitable for analysis. The remaining audio files were not available for analysis due to equipment failure (*n* = 10), session leaders not recording the sessions (*n* = 17), and sound problems that led to poor quality audio files (*n* = 2). Audio recording of health behaviour maintenance sessions indicated a mean session length of 24.6 min (SD = 16.74) compared to the planned 45 min. Table 4 shows characteristics of the sessions analysed (intervention group, intervention site, intervention provider, participant numbers and proportion of sampled sessions analysed).

**Table 4:** Characteristics of sessions sampled

Intervention Group	Intervention Site	Intervention Provider	Session Leader	N of participants	N of sessions sampled	N of sessions recorded (%)	N of sessions used in analysis (% of sampled sessions)
Group 1	Bristol/Bath	Provider 1	F1	13	9	8 (89)	6 (67)
Group 2	Bristol/Bath	Provider 2	F2	15	9	6 (67)	4 (44)
Group 3	Bristol/Bath	Provider 3	F1	16	9	7 (78)	7 (78)
Group 4	Birmingham	Provider 4	F3	15	9	6 (67)	2 (22)
Group 5	Bristol/Bath	Provider 2	F4	14	9	5 (56)	5 (56)
Group 6	Devon	Provider 5	F5	3	9	2 (22)	1 (11)

### Intervention delivery fidelity

The overall delivery fidelity for the intervention (the mean of the scores for each intervention BCT, taken across all groups at all sites) was 2.5 (SD 0.45), indicating that, overall, intervention delivery fidelity was sub-optimal (Table 5). The overall fidelity broken down by group was broadly similar, with mean intervention scores ranging between 2.4 and 2.9. However, one group (Group 4) had consistently lower delivery fidelity scores (Mean 1.7).

**Table 5:** Overall delivery fidelity scores for intervention BCTs and processes

Group	Facilitator ID	Person-Centred Delivery	Facilitating Enjoyment	Monitoring Progress Acknowledge and Review	Monitoring Progress Eliciting benefits of PA	Self-Monitoring	Managing Setbacks and Problem-solving	Goal setting and action planning	Modelling	Promoting Autonomy	Supporting Self-Efficacy for PA	Supporting Relatedness	Overall Score
1	F1	3.3	3.5	2.2	1.8	3	1.9	1.8	3	3	2.2	2.2	**2.5**
2	F2	2.6	2.8	2.6	1.5	3	1	2.6	3	2.4	1.9	2.9	**2.4**
3	F1	3.4	3.5	3.3	2	4	2.6	2.5	3	3	2.7	2.4	**2.9**
4	F3	2.5	2.5	2	2	1	1	0	2	2.5	2	1	**1.7**
5	F4	3.2	3.2	2.6	2.2	3	1.9	3	3	3.2	2.9	2.5	**2.8**
6	F5	4	3	3.5	2.5	3	3	3	0	4	2.5	2	**2.8**
**Mean Scores**		**3.2**	**3.1**	**2.7**	**2**	**2.8**	**1.9**	**2.2**	**2.3**	**3**	**2.4**	**2.2**	**2.5**
**Standard Deviation**		**0.55**	**0.39**	**0.59**	**0.34**	**0.98**	**0.81**	**1.14**	**1.21**	**0.57**	**0.39**	**0.65**	**0.46**

The fidelity scores for each BCT and delivery process are outlined in Table 5 and the raw fidelity scores are shown in Additional file 5. Three BCTs/processes; Person-centred delivery, Facilitating Enjoyment, and Promoting Autonomy were scored as having competent delivery fidelity. Six BCTs/ processes; Monitoring progress (acknowledging and reviewing), Self-Monitoring, Monitoring progress (eliciting benefits of PA), Goal Setting and Action Planning, Modelling, Supporting Self-Efficacy for PA, and Relatedness were scored from 2.0 to 2.9, indicating scope for improvement of delivery fidelity. One BCT – Managing Setbacks and Problem Solving had a low delivery fidelity (Mean 1.9, SD 0.81). A detailed list of good practice and practice requiring improvement, associated with each BCT or process is provided in Additional file 4.

### Overall intervention fidelity across groups

**Fig. 2:**
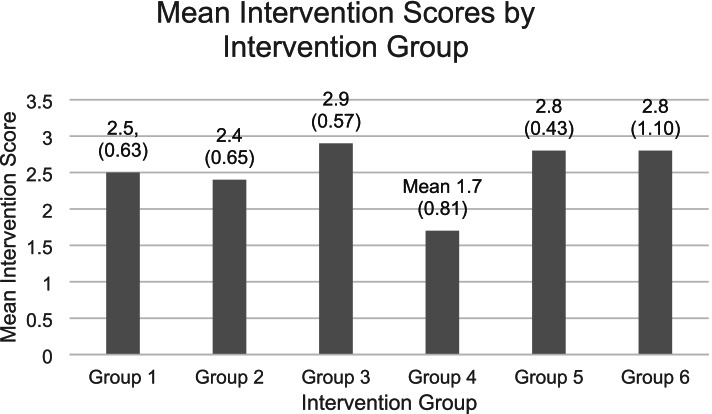
Mean (SD) Intervention score by intervention group

The overall delivery fidelity scores for each intervention group are shown in Fig. 2. Scope for improvement was reported for Group 1 (Mean 2.5, SD = 0.63), Groups 3 (Mean 2.5, SD = 0.57), Group 6 (Mean 2.8, SD = 1.10), Group 5 (Mean 2.8, SD = 0.43) and Group 2 (Mean 2.4, SD = 0.65). Low delivery fidelity was reported for Group 4 (1.7; SD = 0.81).

### Examples of REACT delivery practice

A wide range of examples were identified of both ‘good practice’ and practice requiring improvement, observed in delivery of each intervention checklist item. A full list is provided in Additional file 4.

## Discussion

The overall mean score for intervention delivery fidelity (2.5, SD = 0.45) indicated that, on average, across the sample studied, there was scope for improvement in the delivery of the behavioural and maintenance support components of the REACT intervention. There were several examples of good practice, but also several examples of practice requiring improvement and practice that contradicted the intended delivery model. There was considerable variation in delivery fidelity between intervention BCTs and processes, between session leaders and between intervention groups. Key BCTs needing improvement of delivery fidelity included Monitoring progress (eliciting benefits of PA), Goal Setting and Action Planning, Modelling, Supporting Self-efficacy for PA, Supporting Relatedness. A key BCT scoring low delivery fidelity was Managing Setbacks and Problem Solving. The variation in delivery fidelity between groups illustrates the importance of ensuring consistency of delivery fidelity in group-based interventions, as poor facilitation in one group or centre could undermine a) effectiveness for participants of that group (up to 15 per REACT group) and b) effectiveness of the entire intervention.

The current study adds to an emerging body of work on intervention fidelity [11, 20–23]. It is consistent with this evidence in finding that the quality in the delivery of complex behavioural interventions varies considerably between session leaders and from group to group. The inter-group variation in fidelity may reflect variations in intra-group dynamics, so teaching skills for managing these dynamics could be an important consideration for future training of intervention facilitators [24]. Other studies have used a mixed-methods approach (interviews alongside session observation) to assess fidelity of exercise session delivery [23]. Hence, in future research, it may be possible to combine fidelity analysis of both exercise and behavioural /education components for multi-modal programmes like REACT.

Reasons for lower delivery fidelity varied from incomplete delivery of BCTs or processes to missing opportunities to deliver a BCT or process. In some instances, BCTs were delivered, but there was little adaptation for different contexts, or checking for participant understanding, or summarising of discussions. This could be due to a lack of experience in using the intended BCTs or processes or alternatively poor performance could be due to lack of engagement skills and inability to facilitate a wider discussion on the topic. Time constraints are also a potential reason for lower delivery fidelity of BCTs. The presence of time-constraints was also implied by the mean session delivery time of 24.6 min compared with the intended 45 min.

A systematic review of physical activity interventions in older adults, which assessed associations between intervention effectiveness and the use of specific BCTs, suggested that some self-regulation techniques may not be acceptable to older adults [25, 26]. This may be because they are less likely to be concerned with attaining a particular level of physical activity and more concerned with the associated enjoyment and social connectedness of the group experience [25–27]. Further evidence of the poor acceptability of self-regulatory BCTs comes from a recent qualitative study which suggested age-related cognitive decline could play a role in reducing acceptability and the effectiveness of self-regulatory BCTs [28]. Hence, the sub-optimal delivery of self-regulation techniques in the REACT study may, to some extent, reflect resistance to such techniques by the participants, which the session leaders responded to by downplaying these elements of the intervention. Participant “pushback” has been reported as a factor in lower delivery fidelity for physical activity-related BCTs in an intervention using physical activity to assist smoking reduction [21]. Taken together, these studies suggest that participant engagement with BCTs could play an important role in delivery fidelity [29].

As well as self-regulation, low scores for the social processes of Supporting Relatedness and Modelling were observed. As such, important elements of the intervention’s underlying theory (SDT and SCT) were not proactively delivered by session leaders [30, 31]. Despite this, it may be the case that participants gained significant encouragement and motivation from social interactions and mutual support within the group setting [24]. It is important to stress that fidelity of delivery was only assessed in the health behaviour maintenance sessions and not in the exercise sessions. In-vivo observation of some of the REACT intervention exercise sessions suggested that there was evidence that session leaders actively supported processes such as modelling and relatedness throughout delivery of the exercise component of the intervention. Furthermore, when considering the time afforded to the health behaviour maintenance sessions, exercise professionals may have viewed their primary role as delivering the exercise session, which they may have been more competent in delivering anyway, with the health behaviour maintenance session being supplementary.

### Strengths and limitations

Assessing intervention fidelity using coding of audio-recorded intervention delivery sessions is considered a gold standard method [8]. Although time-consuming and labour-intensive, this method allowed direct observation of intervention delivery and an assessment that was specifically tailored to the REACT intervention and its associated logic model (Fig. 1). Scoring was based on a validated response scale designed for coding the acquisition of skills and reliability was enhanced by using independent coders for the first 10 sessions to calibrate the coding and minimise subjective bias. The notes taken during coding of the recordings allowed the gathering of examples of both good delivery practice and delivery practice needing improvement. This both added richness to the quantitative assessment and provided real-life examples and scenarios that can be used (as a basis for discussion, practice exercises, or illustration of good practice) in future REACT facilitator training. A further strength of this study was the sampling of recordings across a diverse range of intervention BCTs and a diverse range of REACT intervention sites, session leaders and intervention providers.

Limitations of this study include a relatively small sample size, a common limitation in fidelity research [32], which was not sufficient to allow mediation analysis exploring whether variations in fidelity scores affected REACT intervention outcomes. There is also potential for sampling bias, given that we were only able to score fidelity for 25 out of our intended sample of 54 sessions. This may have led to over-estimation of intervention fidelity if the recordings were more likely to be missing at sites where performance was low. In addition, low availability of data prevents robust comparison of fidelity data between groups, highlighting the importance of establishing a threshold for the percentage of sessions sampled, a limitation of this study. Future fidelity studies should consider establishing such a threshold to reduce the risk of sampling bias.

Furthermore, the rating approach used was subjective, so there is no definitive way to ensure that a score of 3 truly represents “competent” delivery. Despite this, raters were confident that there was clear identification of areas needs for improvement in the delivery of the health behaviour maintenance sessions and the process yielded clear ideas for how intervention delivery can be improved. In addition, the iterative calibration approach used for checking coding reliability did not allow for testing of inter-rater reliability. However, an additional post-calibration check on inter-rater reliability could be included in future intervention fidelity studies.

### Recommendations for practice

Future training of REACT session leaders should include examples of competent delivery that have been identified in this fidelity study (Additional file 4). Future REACT training courses should particularly focus on the BCTs and processes that were identified here as having sub-optimal delivery fidelity. Involving session leaders and participants in the refinement of the health behaviour maintenance sessions and translation of theoretical constructs and BCTs into deliverable sessions may lead to better delivery fidelity [33, 34]. In addition, trainees should be given the opportunity to practice delivery of BCTs and have this overseen by professionals with suitable experience in the delivery of relevant BCTs.

Given the variation in delivery fidelity observed here, high quality training and quality assurance processes may be crucial to ensure the effectiveness of the intervention when transitioning from the context of a research study to wider scale community-based implementation. This might, for example, involve rating of delivery fidelity for each trainee post-training (by independent observation or self-rating), performance monitoring, or other methods for identifying ongoing training needs. Booster sessions could be offered throughout the intervention as a means of maintaining trainer competence and confidence in delivery of BCTs. Time pressures on delivery might be addressed by systems-level interventions involving the manipulation of reward criteria by funders or improving internal governance /quality assurance procedures within provider organisations.

### Recommendations for future research

The potential benefit of teaching techniques and skills for promoting positive intra-group dynamics /mutual support for improving the delivery of the intended intervention processes should be explored in further research. The impact of participant reactions to BCTs or “pushback” on delivery fidelity should also be explored.

Considering fidelity data alongside qualitative data from facilitator and participant interviews, as well as quantitative process data would add depth and rigour through the triangulation of data from different sources [20, 32, 35–37]. For example, data from facilitator interviews would allow exploration of possible reasons for low delivery fidelity of BCTs and the challenges faced by session leaders in delivering the intended programme. Data from interviews with participants could lead to a better understanding of variations in receipt, enactment and intervention outcomes [10]. Using questionnaires to measure changes in the intended psychosocial /cognitive targets of the intervention such as self-efficacy, autonomy and relatedness across the whole sample would allow fidelity data to be related to intervention effects on these measures.

## Conclusions

There is a clear scope for improvement in the delivery of both self-regulation processes and social /relatedness-building processes within the REACT intervention. There is also a need to improve the consistency of delivery among session leaders and among groups. Our synthesis of the findings generated several recommendations for future intervention delivery. The integration of fidelity assessment into intervention design and delivery, involving exercise session leaders in the intervention design, and conducting mixed-methods process evaluations has the potential to inform the iterative improvements in the content and effectiveness of behaviour change interventions promoting physical activity.

## Supplementary Information

**Additional file 1.** Sample REACT Health Behaviour Maintenance Session Plan.

**Additional file 2.** Fidelity Measure Scoring Instructions.

**Additional file 3.** Scoring of REACT Intervention BCTs and processes on the 11-item fidelity checklist.

**Additional file 4.** Examples of REACT Delivery Practice.

**Additional file 5.** Raw data for intervention fidelity scores.

## Data Availability

The raw data scores for intervention fidelity components are available in Additional file 5. The audio data from which these scores are derived cannot be shared publicly, as they are not anonymised. Participants may refer to people or places that could lead to their identification, or identification of the intervention provider. The research funding does not allow for transcribing and anonymising of this large body of audio data.

## Abbreviations

BCC: Behaviour Change Consortium
BCTs: Behaviour Change Techniques
PA: Physical Activity
RCT: Randomised Controlled Trial
REACT: REtirement in ACTion
SCT: Social Cognitive Theory
SDT: Self-Determination Theory
