# Supplementary material for: Delivery fidelity of the REACT (REtirement in ACTion) physical activity and behaviour maintenance intervention for community dwelling older people with mobility limitations
Source: BMC Public Health. 2022 Jun 3;22:1112. doi: 10.1186/s12889-022-13496-z (PMC9166457; doi:10.1186/s12889-022-13496-z)
Supplement: Supplementary file 1 — Additional file 1. Sample REACT Health Behaviour Maintenance Session Plan. [file 12889_2022_13496_MOESM1_ESM.docx]

**Additional File 1**

**Sample REACT Health Behaviour Maintenance Session Plan**

**Week 48 Health Behaviour Maintenance Session
Use it or Lose it 3!**

| **OBJECTIVES** |
| --- |
| - To discuss the details of participants’ action plans and their commitment to making it work - Focus on detail of how individuals are going to make their plan work - To encourage participants to include other group members in their plans - Encourage people to get started on the plan before REACT finishes |
| **KEY MESSAGES** |
| - Having a plan can help you meet your fitness goals - Finding activities that you find enjoyable will make it easier to keep going - Planning activities with other people or members of the group makes things more fun and makes it more likely you will attend - If you can’t exercise for a while have a plan about how you can get started again |
| **RESOURCES** |
| - 15 chairs - Coffee /tea making facility for 17 people (15 + staff) - Register - Flip chart, markers (or large piece of flipchart paper, pens and use of a large table) - Digital recorder - Hand-outs: Action plans |

**N.B 3 days before session, auto-text to remind participants to bring their completed action plans with them.**

**SESSION OVERVIEW**

| **time** | **Topic / Activity** | |
| --- | --- | --- |
| 10 | Tea & Chat | |
| 10 | Our progress | |
| 25’ | Use it or Lose it 3 (topic of the day) | |
| **45’** | **Total** |  |

**DETAILED PLAN**

| **Time** | **Topic / Activity** | **Resources** |
| --- | --- | --- |
| 10’ | Tea and chat as usual | - Tea /coffee /mugs or disposable cups |
|  | **Start audio recording if applicable (check there are no objections from the group)** | - Digital recorder |
| **10** | **Our progress:**   - ASK: Let’s review your pedometer reports. Did you see a difference in your total steps as a result of trying out new activities? - ASK: Let’s talk about any new activities you’ve started doing - TELL: Celebrate any successes (e.g. Well-done /great progress). It is normal that some of us met this goal and some did not. That’s why we are all here. To support each other in the REACT journey. |  |

| 25 | **Use it or Lose it 3 (topic of the day)**   - TELL: Recap on discussion from Week 44 - ASK: Give out fresh activity menus (from WEEK 40) – ask people who have not completed plans to look at the menus and have a think/to complete their plans by next time. - TELL; Repeat instructions from last time about filling in the plans - DISCUSS: What kind of things are people planning to do? Elicit ideas and use the opportunity to give a bit of individual support (strategic reflective listening here to reinforce positive ideas and suggest possible extensions, or tweaks that will add value in terms of a) checking the intensity of activities b) making them more fun c) making them more socially engaging ) – this will reward people for completing the task - ASK: Create an updated “menu” of things that are happening in the local area – by asking people about activities they have attended or heard about - DISCUSS: Recap on how to get back on the wagon if you have a health problem that stops you exercising for a few weeks, or even more? (basically, it is like starting the REACT programme again – however, it should only take about 8 weeks to get back to your best fitness level – need to know where you can book sessions (or can do same exercises we did here at home, OR can use an exercise DVD /online exercise programme on a tablet or laptop. If possible provide a handout on recommended (ideally free, or low cost) options? - DISCUSS: Feel free to get together in pairs or groups of 3 or more between now and next time to make a joint plan of things that you can do together – we strongly encourage this, but it is up to you to arrange to meet up and do this |  |
| --- | --- | --- |

|  | PREPARE FOR NEXT SESSION:  **TELL:** Please bring your plans back next time – and tell us about what progress you have made in achieving them  **ASK:** Would you like to bring in some food or snacks for our last session in 4 weeks’ time to celebrate a whole year of taking part in the REACT study?  **CLOSE** |  |
| --- | --- | --- |
