# Supplementary material for: Delivery fidelity of the REACT (REtirement in ACTion) physical activity and behaviour maintenance intervention for community dwelling older people with mobility limitations
Source: BMC Public Health. 2022 Jun 3;22:1112. doi: 10.1186/s12889-022-13496-z (PMC9166457; doi:10.1186/s12889-022-13496-z)
Supplement: Supplementary file 2 — Additional file 2. Fidelity Measure Scoring Instructions. [file 12889_2022_13496_MOESM2_ESM.docx]

**Additional File 2**

**Fidelity Measure Scoring Instructions**

The scoring instructions for the fidelity checklist used to assess delivery fidelity of REACT intervention processes and BCTs is detailed below.

**The Dreyfus Scale**

The six-point rating scale is based on the Dreyfus system for denoting skill and competence and is intended to measure the session leader’s adherence to intervention behaviour change techniques (BCT) as intended, as well as the skill with which they are delivered. The six point scale ranges from (0) where the session leader hasn’t delivered the intervention BCT appropriately, whether it was delivered poorly or insufficiently (low fidelity) to (5) where the session leader has delivered the BCT appropriately, sufficiently or with a high degree of skill (high fidelity). To help with the scoring of session leaders use of intervention BCTs and intervention processes, an outline of the key features of each BCT and intervention process is provided. It should be noted that the giving a 5 (expert) should be reserved for session leaders who deliver BCTs or intervention processes exceptionally well, adapting for different contexts and often in the face of difficulties such as resistance from participants. In the instance that the session leader experiences difficulties (resistance from participants etc) the session leader should be assessed on their delivery of the BCT or intervention processes even if this fails to lead to a change in the participant’s behaviour, it should be the session leaders skill of delivery and interaction with the participant that should be assessed.

**Scoring Facilitator Delivery Fidelity**

When rating the session leader’s skill at delivering a particular BCT or intervention process, first identify if the key features of the item is evident. Then consider if the key features were used appropriately (misses few opportunities to deliver and when doing so, delivers them well). In this instance the session leader should be rated highly. It should be noted that the scores produced from using this fidelity measure should follow a roughly normal distribution, with relatively few session leaders scoring at the extremes of the scales (low/high scores). However, this may be skewed by consistently good or poor delivery for a particular session leader. For the purpose of the REACT study, a score of 3 or higher will indicate the session leader has demonstrated competent delivery (in the view of the intervention designers).

**Person-centred delivery**

**Key features**: Communication between the session leader and participants should be participant-centred, to encourage participants to be the main agent of change; maximising their autonomy and sense of intrinsic motivation. The session leader should empower the participant to provide input and choose the ways in which they progress.

**Delivery****:** Session leaders should use OARS (Open ended questioning, Affirmation, Reflective listening, Summaries). Open ended questions would involve the session leader allowing the participants to express their feelings creatively, revealing participant thought process and points of reference. Affirmations or praise should be given for positive behaviours, recognising the participant’s efforts towards change, including change talk (commitment, desire, ability to change, reasons to change, or need to change) as well as their agency or autonomy in making any changes. Reflective listening would involve actively engaging with and reflecting on participant contributions. Session leaders should use this technique to direct further conversation or highlight key strengths and barriers previously discussed. Summaries can be used to reinforce participant choices and acknowledging participant effort or success. The Ask-Tell-Discuss technique may be used to enhance engagement when exchanging information. The communication style used by session leader should be empathetic and facilitate participant enjoyment and should in no way be didactic.

Table 1. Fidelity Measure Scoring - Person-centred delivery

| **Rating** | **Score** | **Example** |
| --- | --- | --- |
| **0** |  | Absence of person-centred delivery style. An overly ‘didactic’ style of interaction, which could result in participant resistance and or highly inappropriate delivery. |
| **1**  **Novice** |  | Minimal use of person-centred delivery style. An overly didactic style of interaction and or inappropriate delivery |
| **2**  **Advanced Beginner** |  | Some evidence of competence using person-centred delivery style (but these are infrequent), however, these may not be carried out to sufficient detail (missing features, e.g. reflexive listening or summaries). There are numerous problems or inconsistencies such as detached involvement or a facilitator who sometimes dominates the discussion. |
| **3**  **Competent** |  | Competent and appropriate use of person-centred delivery style, however some difficulties are evident (e.g. failing to summarise the discussion). Awareness of and ability to cope with different context and situations but some minor problems or inconsistencies. More engaged involvement from the facilitator. |
| **4**  **Proficient** |  | Numerous and appropriate usage of person-centred delivery style. The session leader encourages the participant to be actively involved and driving the discussion. The session leader is able to discriminate between a variety of contexts and situations with minimal problems or inconsistencies. |
| **5**  **Expert** |  | Highly appropriate and sufficient use of person-centred delivery style. The session leader encourages the participant to be actively involved. Able to make more subtle, refined discriminations between situations and contexts and able to adjust accordingly. No problems or inconsistencies. |

**Facilitating Enjoyment**

**Key features:** Session leaders should focus on making the social interactions positive, supportive and enjoyable, rather than embarrassing and awkward.

**Delivery:** Session leaders should use OARS (Open ended questioning, Affirmation, Reflective listening, Summaries) to encourage engagement and interaction with the group. Open ended questions would involve the session leader allowing the participants to express their feelings creatively, revealing participant thought process and points of reference. Affirmations or praise should be given for positive behaviours or ideas which increase enjoyment or lead to positive social interaction within the group. Reflective listening would involve actively engaging with participants’ “banter” and reflecting on positive participant contributions. Summaries can be used to reinforce participant choices and acknowledging participant effort or success. Session leaders should use the above techniques to encourage a sense of fun and positive social interactions. Session leaders should use their “fun detectors” to join in with and reinforce any positive, good-humoured or interesting interactions. However, they should also reflect on and try to block any discriminatory or demeaning interactions and should ensure that the session content is delivered (avoiding spending too much time on non-relevant content).

Table 2. Fidelity Measure Scoring– Facilitating Enjoyment

| **Rating** | **Score** | **Example** |
| --- | --- | --- |
| **0**  **Absence** |  | Absence of focusing on participant enjoyment, no effort to make interactions positive or fun and/or highly inappropriate delivery |
| **1**  **Novice** |  | Minimal focus on participant enjoyment, minimal effort to make interactions positive or fun and or inappropriate delivery. |
| **2**  **Advanced Beginner** |  | Some evidence of competence and adapting appropriately to the context involved (but these are infrequent). Some focus on enhancing participant enjoyment, however, this may not be carried out in sufficiently or sustained and opportunities may be missed. There are numerous problems or inconsistencies such as detached involvement from the session leader. |
| **3**  **Competent** |  | Competent and appropriate focus on participant enjoyment. Facilitating positive and fun interactions even if difficulties or awkwardness are evident. The session leader is engaged and aware of and able to cope with different contexts and situations but some minor problems or inconsistencies (e.g. a change in mood going unnoticed by the session leader). |
| **4**  **Proficient** |  | Numerous appropriate examples of facilitating participant enjoyment through support of positive and fun interactions. The session leader is able to discriminate between a variety of contexts and situations with minimal problems or inconsistencies. |
| **5**  **Expert** |  | Highly appropriate and sufficient facilitation of participant enjoyment, through support of positive and fun interactions. The session leader is able to make more subtle refined discriminations between contexts and able to adjust accordingly. Minimal problems. Excellent features, no problems or inconsistencies. |

**Monitoring progress – Acknowledge and Review of PA**

**Key features**: Session leaders should discuss progress in the following areas (physical activity (PA) levels, perceived emotional benefits of PA, perceived physical benefits of PA, perceived social benefits of PA). Session leaders should identify the most important points and create a comfortable platform on which the participant can discuss them. Where possible session leaders should acknowledge and appraise participant progress and give feedback in a positive, encouraging manner. Session leaders should encourage participants to start self-monitoring their own physical activity behaviours and any subsequent benefits they experience.

**Delivery****:** The session leader should use all opportunities to acknowledge and reinforce progress and successes with behaviour change. Session leaders should use OARS (Open ended questioning, Affirmation, Reflective listening, Summaries). Open ended questions would involve the session leader allowing the participants to focus on their progress as they experience it. In doing so, revealing participant thought processes and points of reference that drive the discussion. Affirmations or praise should be given for positive behaviours, recognising the participant’s efforts towards increasing physical activity levels (regardless of success), including change talk (commitment, desire, ability to change, reasons to change, or need to change) as well as their agency or autonomy in making any changes. Reflective listening would involve actively engaging with participants and reflecting on participant contributions. Session leaders should use this technique to direct further conversation or highlight key strengths and barriers previously discussed. Summaries can be used to reinforce participant choices and acknowledging participant effort or success. The Ask-Tell-Discuss technique may be used to enhance engagement when exchanging information.

**Monitoring Progress – Acknowledge and Review**

Table 3. Fidelity Measure Scoring – Monitoring Progress – Acknowledge and Review

| **Rating** | **Score** | **Example** |
| --- | --- | --- |
| **0**  **Absence** |  | Absence of discussion to monitor participant progress in physical activity behaviours and/or highly inappropriate performance |
| **1**  **Novice** |  | Minimal discussion to monitor participant progress in physical activity behaviours and/or inappropriate delivery |
| **2**  **Advanced Beginner** |  | Some evidence of competence when discussing participant progress in physical activity behaviours (but these are infrequent) and these may not be carried out to sufficient depth or detail. There are numerous problems and inconsistencies such as detached involvement or missed opportunities to deliver the behaviour change technique. |
| **3**  **Competent** |  | Competent and appropriate attempts to discuss monitoring participant progress in physical activity behaviours. The session leader is engaged and aware of and able to cope with different context and situations but there some minor problems or inconsistencies (e.g. not covering all components of the behaviour change technique to sufficient depth or failing to summarise the discussion). |
| **4**  **Proficient** |  | Numerous appropriate discussions to monitor participant progress in physical activity behaviours, able to discriminate between a variety of contexts and situations with some minor problems or inconsistencies evident. |
| **5**  **Expert** |  | Highly appropriate and sufficient discussions monitoring participant progress in physical activity behaviours. Able to make more subtle refined discriminations between contexts and able to adjust accordingly. Minimal problems. |

**Monitoring Progress – Eliciting Benefits of Physical Activity (PA)**

**Key features:** Session leaders should discuss progress in the following areas (physical activity (PA) levels, perceived emotional benefits of PA, perceived physical benefits of PA, perceived social benefits of PA) and the link between these and the behaviour changes they have made. Session leaders should identify the most important points and create a comfortable platform on which the participant can discuss them. Where possible session leaders should acknowledge and appraise participant progress and give feedback in a positive, encouraging manner. Session leaders should encourage participants to start self-monitoring their own physical activity behaviours and any subsequent benefits they experience.

**Delivery:** The session leader should use all opportunities to acknowledge and reinforce progress and successes with behaviour change. Facilitators should use OARS (Open ended questioning, Affirmation, Reflective listening, Summaries). Open ended questions would involve the session leader allowing the participants to focus on their progress as they experience it. In doing so revealing participant thought processes and points of reference that drive the discussion. Affirmations or praise should be given for positive behaviours, recognising the participant’s efforts to increase levels of physical activity (regardless of success), including change talk (commitment, desire, ability to change, reasons to change, or need to change) as well as their agency or autonomy in making any changes. Reflective listening would involve actively engaging with participants and reflecting on participant contributions. Session leaders should use this technique to direct further conversation or highlight key strengths and barriers previously discussed. Summaries can be used to reinforce participant choices and acknowledging participant effort or success. The Ask-Tell-Discuss technique may be used to enhance engagement when exchanging information.

**Monitoring Progress - Eliciting Benefits of Physical Activity**

Table 4. Fidelity Measure Scoring – Monitoring Progress - Eliciting benefits of Physical Activity

| **Rating** | **Score** | **Example** |
| --- | --- | --- |
| **0**  **Absence** |  | Absence of discussion to elicit and reinforce participant perceived benefits of physical activity and or highly inappropriate delivery |
| **1**  **Novice** |  | Minimal discussion to elicit and reinforce participant perceived benefits of physical activity and or inappropriate delivery. |
| **2**  **Advance Beginner** |  | Some evidence of competence when eliciting and reinforcing participant perceived benefits of physical activity and adapting delivery to context (but these are infrequent) and discussions may not be of sufficient depth or detail. There are numerous problems or inconsistencies such a detached involvement or missed opportunities. |
| **3**  **Competent** |  | Competent and appropriate attempts to discuss eliciting and reinforcing participant perceived benefits of physical activity. The session leader is engaged, aware and able to cope with different context and situations. There are some minor problems or inconsistencies (E.g. not covering all components of the behaviour change technique to sufficient depth or failing to summarise the discussion ). |
| **4**  **Proficient** |  | Numerous appropriate discussions to elicit and reinforce participant perceived benefits of physical activity. The session leader is able to discriminate between a variety of contexts and situations with minimal problems or inconsistencies. |
| **5**  **Expert** |  | Highly appropriate and sufficient discussions to elicit and reinforce perceived benefits of physical activity. The session leader is able to make more subtle refined discriminations between situations and contexts and able to adjust accordingly. No problems or inconsistencies. |

**Self-Monitoring**

**Key features:** Session leaders should discuss participants self-monitoring of physical activity behaviours and self-reflection on the outcomes of this behaviour and how this was achieved. Session leaders should discuss ways in which participants can self-monitor physical activity behaviour; e.g. use of pedometers and give them the relevant opportunities to discuss this.

**Delivery**: The session leader should use all opportunities to acknowledge and reinforce participant attempts to self-monitor physical activity behaviour and subsequent progress they have made. Session leaders should use OARS (Open ended questioning, Affirmation, Reflective listening, Summaries). Open ended questions would involve the session leader allowing the participants to focus on their self-monitoring behaviours and any progress they make as they experience it. In doing so revealing participant thought processes and points of reference that drive the discussion. Affirmations or praise should be given for participant self-monitoring as well as recognising the participant’s attempts or efforts to self-monitor (regardless of success), including change talk (commitment, desire, ability to change, reasons to change, or need to change) as well as their agency or autonomy in making any changes. Reflective listening would involve actively engaging with participants and reflecting on participant experiences of self-monitoring. Session leaders should use this technique to direct further conversation or highlight key strengths and barriers previously discussed. Summaries can be used to reinforce participant choices and acknowledging participant effort or success. The Ask-Tell-Discuss technique may be used to enhance engagement when exchanging information.

Table 5. Fidelity Measure Scoring - Self-monitoring

| **Rating** | **Score** | **Example** |
| --- | --- | --- |
| **0**  **Absence** |  | Absence of discussion on self - monitoring behaviours and participant reflection on progress and or highly inappropriate delivery. |
| **1**  **Novice** |  | Minimal discussion on self - monitoring behaviours and participant reflection on progress and or highly inappropriate delivery. |
| **2**  **Advanced Beginner** |  | Some evidence of competence and adapting appropriately to context involved (but these are infrequent) when discussing participant self - monitoring behaviours and participant reflection on progress. There are numerous problems and inconsistencies such as detached involvement or missed opportunities. |
| **3**  **Competent** |  | Competent and appropriate attempts to discuss self - monitoring behaviours and participant reflection on progress. The session leader is engaged, aware and able to cope with different context and situations. There are however some minor problems or inconsistencies (e.g. not covering all components of the behaviour change technique to sufficient depth or failing to summarise the discussion). |
| **4**  **Proficient** |  | Numerous appropriate discussions on self - monitoring behaviours and participant reflection on progress. The session leader is able to discriminate between a variety of contexts and situations with minimal problems or inconsistencies. |
| **5**  **Expert** |  | Highly appropriate and sufficient discussions on self - monitoring behaviours and participant reflection on progress. The session leader is able to make more subtle refined discriminations between situations and contexts and able to adjust accordingly. No problems or inconsistencies. |

**Managing setbacks and Problem solving**

**Key features:** The session leader should work with the participant to review progress with all planned changes and with achieving the targets set out in the action plan. The session leader should celebrate and reinforce and reflect on any successes achieved. The session leader should encourage discussion of any setbacks or lapses and the patient’s plans should be revised accordingly. When setbacks or lapses occur, session leaders should reframe and normalise setbacks or lapses so rather than being viewed as failures they are opportunities for learning and development. When approaching problem solving session leaders should focus on breaking the problem down into achievable parts, and where appropriate considering the sustainability of any change, the ways in which others can be supportive as well as introducing the idea of using coping plans to avoid setbacks. This may involve identifying behaviours to be undertaken to avoid or reduce stressors.

**Delivery**: The session leader should reinforce any self-monitoring activity and any successes in behaviour change (by giving praise/ using affirmation techniques). Reframing should be used to normalise setbacks and see them as an opportunity to learn from experience (trial and error) rather than as failures. The session leader should use OARS (Open questions, Affirmation, Reflective listening, Summaries) when discussing managing setbacks and problem solving. Open ended questions would involve the session leader allowing the participants to focus on their progress, any setbacks that have occurred or problems they have faced. In doing so, revealing participant thought processes and points of reference that drive the discussion. Affirmations or praise should be given for positive behaviours, recognising the participant’s success as well as, efforts towards change despite setbacks or lapses, including change talk (commitment, desire, ability to change, reasons to change, or need to change) as well as their agency or autonomy in making any changes. Reflective listening would involve actively engaging with participants and reflecting on participant contributions. Session leaders should use this technique to direct further conversation or highlight key strengths and barriers previously discussed. Summaries can be used to reinforce participant choices and acknowledging participant effort or success. Problem solving should also use information exchange (Ask-Tell-Discuss) techniques to identify barriers and explore ways to overcome them. Problem-solving may specifically focus on issues of connectedness (social influences, involvement of others in supporting activities) and sustainability, or on breaking the problem down into more manageable chunks. Goals or action plans should be reviewed and revised if necessary. The Ask-Tell-Discuss technique may be used to enhance engagement when exchanging information.

**Managing Setbacks and Problem Solving**

Table 6. Fidelity Measure Scoring – Managing setbacks and Problem Solving

| **Rating** | **Score** | **Example** |
| --- | --- | --- |
| **0**  **Absence** |  | Absence of discussion to review participant setbacks or to suggest appropriate problem-solving strategies relating to physical activity behaviours and/or highly inappropriate delivery |
| **1**  **Novice** |  | Minimal discussion to review participant setbacks or to suggest appropriate problem-solving strategies relating to physical activity behaviours and/or inappropriate delivery |
| **2**  **Advanced Beginner** |  | Some evidence of competence when discussing participant setbacks or problem-solving strategies relating to physical activity behaviours (but these are infrequent) with some adaptation to context. However, there are numerous problems and inconsistencies such as detached involvement and missed opportunities to deliver the behaviour change technique. |
| **3**  **Competent** |  | Competent and appropriate attempts to review participant setbacks or problem-solving strategies relating to physical activity behaviours. The session leader is engaged, aware and able to cope with different contexts and situations. However, there are some minor problems or inconsistencies (e.g. not covering all components of the behaviour change technique to sufficient depth or failing to summarise the discussion) |
| **4**  **Proficient** |  | Numerous appropriate discussions to review participant setbacks or problem-solving strategies relating to physical activity behaviours. The session leader is able to discriminate between a variety of contexts and situations with some minor problems or inconsistencies evident. |
| **5**  **Expert** |  | Highly appropriate and sufficient review of participant setbacks or problem-solving strategies relating to physical activity behaviours. The session leader is able to make more subtle refined discriminations between contexts and able to adjust accordingly. Minimal problems. |

**Goal setting and action planning**

**Key features:** Session leaders should work with the participants to agree on a verbal goals and action plans for behaviour change and behavioural outcomes. This should include negotiating of goals, goal setting and identifying any barriers that may arise. Session leaders should always acknowledge the participants perspective and look for opportunities to allow participant input, so they can be the agent of change.

**Delivery:** Specific goals may be negotiated /set before action plans are formulated. The session leader should ensure that goals and action plans are realistic. When action planning is discussed, session leaders should ensure participants have opportunities for input and so drive the process themselves. Where action-planning is part of the session plan, the session leader should encourage each person present to make at least one plan. The session leader should, where possible, encourage goals and plans that are specific (e.g. when, what, where, who with) and achievable. The session leader may also use some problem-solving examples and techniques at this point to pre-empt and address any foreseeable problems (making a coping plan). When reviewing goals and action plans session leaders should draw attention to discrepancies between current behaviours and goals or action plans and work collaboratively to review the goals and action plans set previously. The action plan menu and “activity snacks” activity should be used (see session plans for Week 11) to facilitate activity planning. This should be achieved using OARS (Open questions, Affirmation, Reflective listening, Summaries). Open ended questions would involve the session leader allowing the participants to focus on their own goals and plans to achieve them. In doing so, revealing participant thought processes and points of reference that drive the discussion. Affirmations or praise should be given for positive behaviours, recognising the participant’s success as well as efforts towards change, including change talk (commitment, desire, ability to change, reasons to change, or need to change) as well as their agency or autonomy in making any changes. Reflective listening would involve actively engaging with participants and reflecting on participant contributions. Session leaders should use this technique to direct further conversation or highlight key strengths and barriers previously discussed. After which the session leader should summarise what was discussed. to reinforce participant choices and acknowledge participant effort or success thus far.

Table 7. Fidelity Measure Scoring - Goal setting and action planning

| **Rating** | **Score** | **Example** |
| --- | --- | --- |
| **0**  **Absence** |  | Absence of attempts to encourage goal setting and action-planning or highly inappropriate delivery. |
| **1**  **Novice** |  | Minimal attempts to encourage goal setting and action-planning and or inappropriate delivery. |
| **2**  **Advanced Beginner** |  | Some evidence of competence and adapting appropriately to the context involved when discussing goal setting and action-planning (but these are infrequent) and may not be carried out to sufficient detail. There are numerous problems and inconsistencies such as detached involvement or missed opportunities to deliver the behaviour change technique |
| **3**  **Competent** |  | Competent delivery and appropriate attempts to encourage goal setting and action-planning. The session leader is engaged, aware and able to cope with different contexts and situations but there are some minor problems or inconsistencies (e.g. not covering all components of the BCT to sufficient depth or failing to summarise the discussion). |
| **4**  **Proficient** |  | Numerous appropriate attempts to encourage goal setting and action-planning. The facilitator is able to discriminate between a variety of contexts and situations with minimal problems or inconsistencies. |
| **5**  **Expert** |  | Highly appropriate and sufficient attempts to encourage goal setting and action-planning. The facilitator is able to make more subtle, refined discriminations between situations and contexts and able to adjust accordingly. No problems or inconsistencies. |

**Modelling**

**Key Features:** Session leaders should give participants the opportunities to observe others engaging appropriately and successfully with the programme (in terms of increasing their physical activity as well as engaging with goal-setting and action-planning, self-monitoring, supporting each other and other behaviour change processes); in doing so participants can acquire new skills and behaviours, especially if they identify positively with the model.

**Delivery:** Session leaders should identify participants succeeding with techniques and behaviours and reinforcing this with positive feedback, affirmation and bringing it to the attention of the group, as an opportunity to observe success. Session leaders should use OARS to communicate this in a positive and sensitive manner to avoid embarrassment (Open ended questioning, Affirmation, Reflective listening, Summaries). Open ended questions would involve the session leader allowing the participants to express their feelings about their successful engagement with the programme so that this can be the focus of the discussion. Affirmations or praise should be given for positive behaviours, recognising the participant’s success as well as efforts towards change, including change talk (commitment, desire, ability to change, reasons to change, or need to change) as well as their agency or autonomy in making any changes. Reflective listening would involve actively engaging with and reflecting on participant contributions. Session leaders should use this technique to direct further conversation or highlight key strengths and barriers previously discussed. Summaries can be used to reinforce participant choices and acknowledging participant effort or success.

Table 8. Fidelity Scoring Measure - Modelling

| **Rating** | **Score** | **Example** |
| --- | --- | --- |
| **0**  **Absence** |  | Absence of modelling used by session leaders to give participants the opportunity to observe others and or highly inappropriate delivery. |
| **1**  **Novice** |  | Minimal use of modelling by session leaders to give participants the opportunity to observe others and or inappropriate delivery. |
| **2**  **Advanced Beginner** |  | Some evidence of competence and adapting appropriately to context involved when using modelling (but this is infrequent) and these may not be carried out to sufficient detail. There are numerous problems and inconsistencies such as detached involvement or missed opportunities. |
| **3**  **Competent** |  | Competent delivery and appropriate use of modelling by session leaders to give participants the opportunity to observe others. The session leader is engaged, aware of and able to cope with different contexts and situations. However, there are some minor problems or inconsistencies (E.g. not covering all components of the BCT to sufficient depth or failing to summarise the discussion) |
| **4**  **Proficient** |  | Numerous appropriate use of modelling by session leaders to give participants the opportunity to observe others. The session leader is able to discriminate between a variety of contexts and situations with minimal problems or inconsistencies. |
| **5**  **Expert** |  | Highly appropriate and sufficient use of modelling by session leaders to give participants the opportunity to observe others. The session leader is able to make more subtle refined discriminations between situations and contexts and able to adjust accordingly. No problems or inconsistencies. |

**Promoting Autonomy**

**Key features:** Session leaders should encourage the participants to be pro-actively (rather than passively) involved in discussions. The aim is to maximise the participants autonomy by developing intrinsic rather than extrinsic motivation, by encouraging them to be the driver of their own success, while developing a sense of control. Session leaders should acknowledge the participants’ perspectives, whilst sharing their own expertise and ideas in a collaborative fashion. Session leaders should always look for opportunities to allow participant input and ideas and choices surrounding physical activity behaviours.

**Delivery:** Session leaders should use OARS (Open ended questioning, Affirmation, Reflective listening, Summaries). Open ended questions would involve the session leader allowing the participants to express their feelings towards topics being discussed so that session leaders can use this as a point of reference to drive discussion forward. Affirmations or praise should be given for positive behaviours, recognising the participant’s success as well as efforts towards change, including change talk (commitment, desire, ability to change, reasons to change, or need to change) as well as their agency or autonomy in making any changes. Where possible session leaders should use reflective listening, which may include reflecting on key points in conversations or may go further to direct the conversation or highlight participants’ strengths or agency in developing plans and overcoming barriers they face. Session leaders should use summaries to reinforce participant decisions and acknowledge efforts, behaviours or achievements made by participants especially if these are desirable. In particular, they should reinforce the fact that any changes made are the participants’ choice. Session leaders should aim to place these techniques in the context of the participants’ individual experiences, knowledge, skill set and current physical activity levels. The Ask-Tell-Discuss technique should be used frequently for exchanging information (to avoid didactic information-giving).

Table 9. Fidelity Measure Scoring - Promoting Autonomy

| **Rating** | **Score** | **Example** |
| --- | --- | --- |
| **0**  **Absence** |  | Absence of attempts to promote autonomy and choice and or highly inappropriate delivery. |
| **1**  **Novice** |  | Minimal attempts to promote autonomy and choice and or inappropriate delivery. |
| **2**  **Advanced Beginner** |  | Some evidence of competence and adapting appropriately to the context when attempting to promote autonomy or choice (but this is infrequent), and these may not be carried out to sufficient depth or detail. There are numerous problems or inconsistencies such as detached involvement and missed opportunities). |
| **3**  **Competent** |  | Competent delivery and appropriate attempts to promote autonomy or choice. The session leader is engaged, aware and able to cope with different contexts and situations but there are some minor problems or inconsistencies (e.g. not covering all components of the intervention process to sufficient depth or failing to summarise the discussion). |
| **4**  **Proficient** |  | Numerous appropriate attempts to promote autonomy or choice. The session leader is able to discriminate between a variety of contexts and situations with minimal problems or inconsistencies. |
| **5**  **Expert** |  | Highly appropriate and sufficient attempts to promote autonomy or choice. The session leader is able to make more subtle, refined discriminations between situations and contexts and able to adjust accordingly. No problems or inconsistencies. |

**Supporting Self efficacy for physical activity (PA)**

**Key features:** Self-efficacy for PA is essential to participants feeling able to enact techniques and behaviours outside of the REACT setting. Session leaders should seek to support self-efficacy for PA by encouraging participants, identifying and breaking down barriers to change, setting achievable goals. Session leaders should encourage gradual and sustainable progress, give appropriate and constructive feedback, encourage problem-solving and ascertain participant confidence and skills so these can be built upon throughout the intervention sessions.

**Delivery**: Session leaders should use OARS (Open ended questioning, Affirmation, Reflective listening, Summaries). Open ended questions would involve the session leader allowing the participants to express their feelings towards topics being discussed so that session leaders can use this as a point of reference to drive discussion forward. Affirmations or praise should be given for positive behaviours, recognising the participant’s success as well as efforts towards change, including change talk (commitment, desire, ability to change, reasons to change, or need to change) as well as their agency or autonomy in making any changes. Reflective listening would involve actively engaging with participants and reflecting on participant contributions. Session leaders should use this technique to direct further conversation or highlight key strengths and barriers previously discussed. Summaries can be used to reinforce participant choices and acknowledging participant effort or success. The Ask-Tell-Discuss technique may be used to enhance engagement when exchanging information and to offer encouragement and acknowledgement of achievements in success and encouragement and constructive feedback to reframe efforts seen as failure. Session leaders should break down goals into manageable chunks and regularly check in with and build upon existing and skills. The aim is to gradually build skills and competence in ability to increase physical activity, by breaking down barriers to change and promoting a positive approach to managing setbacks (i.e. to see them as a learning opportunity, rather than failure).

Table 10. Fidelity Measure Scoring - Supporting Self-efficacy for physical activity

| **Rating** | **Score** | **Example** |
| --- | --- | --- |
| **0**  **Absence** |  | Absence of attempts to build and reinforce self-efficacy for PA and or highly inappropriate delivery. |
| **1**  **Novice** |  | Minimal attempts to build and reinforce self-efficacy for PA and or inappropriate delivery. |
| **2**  **Advanced Beginner** |  | Some evidence of competence and adapting appropriately to the context involved when supporting self-efficacy for PA (but these are infrequent) and may not be carried out to sufficient depth or detail. There are numerous problems and inconsistencies, such as detached involvement or missed opportunities to deliver the intervention process. |
| **3**  **Competent** |  | Competent delivery and appropriate attempts to build and reinforce self-efficacy for PA. The session leader is engaged and aware or and able to cope with different contexts and situations but there are some minor problems or inconsistencies (e.g. not covering all components of the intervention process to sufficient depth or failing to summarise the discussion). |
| **4**  **Proficient** |  | Numerous and appropriate attempts to support self-efficacy for PA. The session leader is able to discriminate between a variety of contexts and situations with minimal problems or inconsistencies. |
| **5**  **Expert** |  | Highly appropriate and sufficient attempts to support self-efficacy for PA. The session leader is able to make more subtle, refined discriminations between situations and contexts and able to adjust accordingly. No problems or inconsistencies. |

**Relatedness**

**Key features**: Fulfilling the need for relatedness (social engagement, social acceptance, peer approval of one’s behaviour and giving support to others) is a key factor in motivating people to initiate and sustained behaviour change. This can be achieved through engagement in physical activity, where there are opportunities for positive social interaction. Session leaders should promote physical activities as opportunities for social connectedness and relatedness.

**Delivery:** OARS (Open questions, Affirmation, Reflective listening, Summaries) and the Ask-Tell-Discuss technique should be used when engaging with participants. Open ended questions would involve the session leader allowing the participants to express their feelings creatively, revealing participant thought process and points of reference. Affirmations or praise should be given for positive behaviours, recognising the participant’s efforts towards change, including change talk (commitment, desire, ability to change, reasons to change, or need to change) as well as their agency /autonomy in making any changes. Reflective listening would involve actively engaging with and reflecting on participant contributions. Session leaders should use this technique to direct further conversation or highlight key strengths and barriers previously discussed. Summaries can be used to reinforce participant choices and acknowledging participant effort or success. The above techniques should be used to create an enjoyable environment where social interaction can take place. Session leaders should be inclusive and welcoming and praise and reinforce positive interactions within the group. Session leaders should aim to make interactions fun and work to diffuse or prevent negative social interactions (e.g. demeaning behaviour /criticism). Session leaders should also encourage people to consider activities that maximise social engagement (e.g. including friends family and spouses and joining group-based activities). In reviewing progress, session leaders should actively seek to encourage reflection on the social benefits of physical activity. Finally, the session leader should help participants to plan to engage social support around engaging in physical activity and to identify and address any negative social influences or social barriers to physical activity.

Table 11. Fidelity Measure Scoring - Relatedness

| **Rating** | **Score** | **Example** |
| --- | --- | --- |
| **0**  **Absence** |  | Absence of attempts to foster relatedness and address social influences and or highly inappropriate delivery. |
| **1**  **Novice** |  | Minimal of attempts to foster relatedness and address social influences and or inappropriate delivery. |
| **2**  **Advanced Beginner** |  | Some evidence of competence and adapting appropriately to the context involved when fostering relatedness (but these are infrequent) and may not be carried out to sufficient depth or detail. There are numerous problems and inconsistencies such as detached involvement or missed opportunities to deliver the intervention process. |
| **3**  **Competent** |  | Competent delivery and appropriate attempts to foster relatedness and address social influences. The session leader is engaged, aware of and able to cope with different contexts and situations but there are some minor problems or inconsistencies (e.g. not covering all components of the intervention process to sufficient depth or failing to summarise the discussion). |
| **4**  **Proficient** |  | Numerous appropriate attempts to foster relatedness and address social influences. The session leader is able to discriminate between a variety of contexts and situations with minimal problems or inconsistencies. |
| **5**  **Expert** |  | Highly appropriate and sufficient attempts to foster relatedness and address social influences. The session leader is able to make more subtle, refined discriminations between situations and contexts and able to adjust accordingly. No problems or inconsistencies. |
