# Supplementary material for: Delivery fidelity of the REACT (REtirement in ACTion) physical activity and behaviour maintenance intervention for community dwelling older people with mobility limitations
Source: BMC Public Health. 2022 Jun 3;22:1112. doi: 10.1186/s12889-022-13496-z (PMC9166457; doi:10.1186/s12889-022-13496-z)
Supplement: Supplementary file 3 — Additional file 3. Scoring of REACT Intervention BCTs and processes on the 11-item fidelity checklist. [file 12889_2022_13496_MOESM3_ESM.docx]

**Additional File 3**

**Scoring of REACT Intervention BCTs and processes on the 11-item fidelity checklist**

For each of the sampled REACT sessions, the scores representing the delivery fidelity for each fidelity checklist item were summarised by calculating either a mean or a maximum score for each item across all sessions. Mean scores were calculated for items representing intervention processes or BCTs that were intended to be delivered in every session (e.g. Person-Centred Delivery and Managing Setbacks and Problem-solving). Maximum scores were used for items representing intervention processes of BCTs that were intended to be delivered in only some of the sessions (e.g. Self-monitoring and Modelling). Table 1. summarises which checklist items were attributed mean or maximum scores.

Table 1. Criteria for summarising scores for each fidelity checklist item

| **Checklist Item** | **Criterion for summarising scores across multiple sessions** |
| --- | --- |
|  |  |
| Person-Centred Delivery Style | Average |
| Facilitating Enjoyment | Average |
| Monitoring Progress | Average |
| Self-monitoring | Maximum |
| Managing Setbacks and Problem-solving | Average |
| Goal setting and action planning | Average |
| Modelling | Maximum |
| Promoting Autonomy | Average |
| Supporting Competence and  Self-efficacy | Average |
| Supporting Relatedness | Average |
