# Supplementary material for: Delivery fidelity of the REACT (REtirement in ACTion) physical activity and behaviour maintenance intervention for community dwelling older people with mobility limitations
Source: BMC Public Health. 2022 Jun 3;22:1112. doi: 10.1186/s12889-022-13496-z (PMC9166457; doi:10.1186/s12889-022-13496-z)
Supplement: Supplementary file 4 — Additional file 4. Examples of REACT Delivery Practice. [file 12889_2022_13496_MOESM4_ESM.docx]

**Additional File 4**

**Examples of REACT Delivery Practice**

A wide range of examples were identified of both ‘good practice’ and practice requiring improvement, observed in delivery of each intervention checklist item. These examples are illustrated below in Table 1.

Table 1. Examples of REACT delivery practice

| **Behaviour Change Technique or process** | **Examples of Good Practice** | **Examples of practice requiring improvement** |
| --- | --- | --- |
| **Person Centred Delivery** | **Group 5, Week 12** The session leader starts a discussion with open ended questions, reflects on the responses of participants, responding where appropriate. The session leader gives options throughout discussion but lets the group direct the conversation. The session leader highlights the group social aspect and that they may enjoy a coffee afterwards. The conversation is natural, and session leader confirms and summaries details at the end before praising them. | **Group 4, Week 9**  The session leader's communication is not participant focused. E.g. asks why the group is quiet, but does not reflect on answers, quickly moving on to ask another question. The session leader frequently talks over participants. |
| **Facilitating Enjoyment** | **Group 3, Week 9** The session leader introduces a name game as means of getting to know each other. The whole group is involved. The facilitator supports the idea by going first, dispelling awkwardness and encourages 'banter' as she goes along. Throughout the game, the session leader praises the group and reinforces positive comments that are made. | **Group 3, Week 12**  The session leader being slow to react when groups were not interacting, or the mood of the group had dropped. |
| **Monitoring Progress - Acknowledge and Review** | **Group 6, Week 16** The session leader asks how the group got on with their 'activity snacks' (a takeaway task). Group discussion ensues with a bit of joking, a positive light-hearted environment in which to discuss progress. One participant reflects on a failure, but the session leader reframes it positively as an improvement asking how the lady felt about it. The session leader and participant move on to discusses a specific goal and action plan for keeping it up and the session leader summarises and confirms with the participant that it is realistic (also good example of realistic and specific goal setting and action planning). | **Group 4, Week 12**  The session leader jokes that a participant was finding it too easy and needed more weight but fails to use it as an opportunity to formally praise them and highlight the progress made. |
| **Monitoring Progress - Eliciting benefits of PA** | **Group 6, Week 16** The session leader praises the group for the progress they've made in increasing activity levels and opens up a discussion about the benefits. The session leader asks participants about the ways they have felt they have benefitted. The session leader’s approach to the discussion is participant focussed in style. | **Group 6, Week 16**  The session leader affirms that they are more active, learning different movements but fails to use the opportunity to explore the benefits of this with the group. |
| **Self-Monitoring** | **Group 3, Week 9** The session leader describes how to use the pedometers to monitor their steps, constantly checking for understanding and clarifying where needed. The session leader describes how the pedometer registers steps and breaks down what she is asking them to do. The session leader praises those who ask questions about the device and highlights barriers they may face and how they might go about overcoming them. The session leader summarises and encourages them to do their best to improve their steps and not to compare efforts to others. | **Group 4, Week 28**  The participant reminds the facilitator to discuss and record their pedometer steps. The session leader records them and asks for them to reset their pedometers to zero for the following week. The session leader offers no praise or feedback on participants’ efforts to self-monitor steps. |
| **Managing Setbacks and Problem Solving** | **Group 3, Week 48** The session leader asks for progress on a participant’s leg injury. Despite the participant's negative outlook on it, the facilitator is positive, reassuring her that her plan of rehabilitation is a good one. The session leader then focusses on the fact that organising a physio appointment is a positive step in the right direction. | **Group 6, Week 16**  The group brings up that they are struggling to exercise in the hot weather, the session leader agrees but doesn't encourage them or offer any ways to overcome this as a barrier. |
| **Goal setting and action planning** | **Group 5, Week 12** The session leader starts a discussion about the goal of doing a group walk with an open-ended question, reflects on the responses of participants, responding where appropriate. The session leader gives options throughout the discussion but lets the group direct the conversation. The session leader highlights the group social aspect and that they may enjoy a coffee afterwards. The conversation is natural, and the session leader confirms and summaries details at the end before praising them. . | **Group 4 Week 12**  A participant starts a conversation about REACT programme completion and what they will do for exercise post-REACT. The session leader misses this opportunity to discuss replacement activities and future action plans. |
| **Modelling** | **Group 1, week 13**  The session leader asks a participant to share their experiences of starting a new activity with the rest of the group. The session leader highlights their success and praises the participant for going. | **Group 3, Week 12**  The session leader highlights a participant's progress and praises them but doesn't use the opportunity to highlight why they are making progress or ask the participant to contribute and reflect on this progress and the way they could be benefitting from it. |
| **Promoting Autonomy** | **Group 5, Week 12** The session leader supports participant choice during a discussion about new activities they are thinking of joining. The session leader offers options for a planned group activity but insists it is 'their choice' and asks open questions about how they feel about this. The session leader lets them discuss and offers help in summarising the discussion asking again how they feel about the particular plan they have formulated. | **Group 5, week 13**  The session leader consistently highlights that participants are able to choose the way in which they complete exercises but doesn't facilitate a discussion about this or encourage participant input. |
| **Supporting Self-Efficacy for PA** | **Group 3, Week 28** Whilst action planning, the session leader asks participants for barriers they face to joining a new class. The session leader breaks each down, offering up solutions that the group goes on to discuss. The interaction is very participant focussed. | **Group 5, Week 13**  The session leader identifies barriers to change but then does not continue the discussion adequately to include breaking down the barriers and seeking the input of participants. |
| **Supporting Relatedness** | **Group 5, Week 12** The session leader supports discussion in organising a group walk, encouraging them to choose their own pace but support each other through it. The session leader continues to encourage a social aspect by organising coffee afterwards. The session leader supports a participant focussed discussion, where most of the interaction occurs between the participants. | **Group 6 Week 16**  A participant discusses problems they experience with their knees and not wanting to drop out of REACT. Another participant offers to work together if they are both limited by mobility, but the session leader fails to encourage this pairing, doesn't use it as an opportunity to discuss the social benefits of physical activity or how they could support each other moving forward. |
